# The immediate effect of COVID-19 pandemic on children and adolescents with obsessive compulsive disorder

**DOI:** 10.1186/s12888-020-02905-5

**Published:** 2020-10-20

**Authors:** J. B. Nissen, D.R.M.A. Højgaard, P. H. Thomsen

**Affiliations:** Department of Child and Adolescent Psychiatry, Aarhus University Hospital, Psychiatry, Aarhus, Denmark

**Keywords:** Obsessive compulsive disorder, Children, Adolescents, COVID-19, Trauma, OCD

## Abstract

**Background:**

Obsessive compulsive disorder (OCD) is a distressing psychiatric disorder. Traumas may trigger or aggravate OCD symptoms. COVID-19 pandemic has coursed a global crisis and has been associated with onset of psychiatric disorders in adults. Little is known about children/adolescents with OCD. The present study aimed to examine how children/adolescents with OCD react towards COVID-19 crisis.

**Methods:**

A questionnaire was distributed to two separate groups of children/adolescents. One group was a clinical group newly diagnosed at a specialized OCD clinic. All the children/adolescents had a current close contact to a therapist or doctor. The other group was a survey group identified through the Danish OCD Association. Most of these children/adolescents were diagnosed years ago, and their primary treatment was completed. For the clinical group, data from patient files was available.

**Results:**

In both groups, but most pronounced in the survey group, participants experienced a worsening of their OCD, anxiety, and depressive symptoms. The aggravation of OCD correlated with the worsening of anxiety, depressive symptoms, and the extent of avoidance behavior. For both groups, OCD aggressive symptoms predicted a significant worsening. Poor baseline insight showed a trend to predict a symptom worsening. The worsening was most pronounced in children with early age of onset and a family history of attention deficit hyperactivity disorder.

**Conclusions:**

To our knowledge, this is one of the first studies examining the effect of COVID-19 in children/adolescents with OCD. The effect was examined in two separate populations strengthening the findings. The study points towards an influence of the OCD phenotype, baseline insight suggesting a continued vulnerability, and a family history of psychiatric disorders.

**Trial registration:**

The study is approved by the Danish Data Protection Agency (1–16–02-147-20) registered 1st of April 2020. Oral and written information was given to parents and patients and written consent from patients over 15 years and parents were received.

## Background

Obsessive compulsive disorder (OCD) is a frequent and distressing psychiatric disorder characterized by unwanted thoughts, images or urges, and repetitive behaviors or mental acts which occur in response to an experienced anxiety or unpleasant feelings. In children and adolescents, OCD often has a major impact on their everyday life [1].

COVID-19 pandemic became a global reality in late winter 2019/spring 2020. The virus spread extensively and fast over borders resulting in a global crisis. The first case of COVID-19 in Denmark was detected on February 27th, 2020, and the first patient died on March 11th. 2020. Thereafter, both Danish and international media gave daily rapports on the COVID-19 situation, the State Health Organization in Denmark created a special home-page as to ensure full and updated information, and the Danish government gave frequent talks about the situation. On March 11th, the Danish government initiated a shutdown of Denmark demanding all public employees with non-essential tasks to work from home, and all schools and day-care centers were closed. Furthermore, gatherings of more than 100, later 10 persons were prohibited. Thus, over a few weeks, children and adolescents were demanded to stay at home, the parents were asked to maintain school work for their children supervised by the teachers on virtual media, most families experienced a feeling of isolation not being able to interact with other people, and children and adolescents were recommended not to meet with more than one friend at a time.

It is known that trauma may be a trigger of OCD symptoms or may contribute to a worsening of symptoms in people already suffering from OCD [2]. Furthermore, OCD symptoms may proceed several years after the epidemic [3]. Several studies have shown a relation between childhood traumas and the severity of anxiety [4], depression [5], psychosis [6] and bipolar disorder [7]. Likewise, a recent study has shown a relation between childhood traumas and the severity of OCD symptoms, the occurrence of comorbid anxiety, increased impulsivity, increased incidence of attention deficit hyperactivity disorder (ADHD) and a lower educational level [8]. Even though fear of COVID-19 infection may not be fully comparable with the above mentioned traumas in childhood, it has been suggested that both depression, anxiety disorders, post-traumatic stress disorder, psychotic disorders and suicide may by consequences of fear of COVID-19, where individuals, who previously have experienced psychological disorders, may be specially prone [9]. Thus, in the early phases of COVID-19, populations of adults in China were asked about how much COVID-19 influenced their lives. Several described the experience of depressive symptoms, anxiety and stress, which were mostly pronounced if they also rated their health as poor [10]. Furthermore, Tanir et al. [11] has shown that youngsters with OCD experienced a significant aggravation of OCD severity, and an increase in contamination obsessions and cleaning/washing compulsions. However, limited research is available on the possible effects of COVID-19 on especially children and adolescents with OCD.

The aims of the present study were to examine the impact of COVID-19 on a clinical population of children and adolescents diagnosed with OCD, and a population of children and adolescents identified through the Danish OCD Association. The clinical group was newly diagnosed at a specialized OCD clinic, and all of them had a current close contact to a psychologist and/or a psychiatrist. Most of them received therapeutic treatment.

The group identified through the Danish OCD Association had been diagnosed years ago, and the primary treatment was completed. This group of prior patients was not necessarily asymptomatic. We wanted to examine OCD symptom severity, and the occurrence or exacerbation of anxiety or depression. Furthermore, we wanted to evaluate if age, gender, previous OCD symptoms, previous OCD symptom severity and treatment effect, comorbidity, previous or current OCD medication, and family psychiatric disorders could predict any changes in OCD symptom severity. We hypothesized that OCD symptom severity would be affected negatively by the COVID-19 crisis, and that children and adolescents with the contamination/cleaning OCD subtype might be particularly sensitive.

## Methods

### Participants

The participants in the study were based on two samples.

The first sample consisted of children and adolescents (7–21 years) newly diagnosed with OCD in a specialised OCD clinic in the Central Denmark Region. The children and adolescents had all been assessed in the specialised OCD clinic using a standardised clinical diagnostic instrument. KSADS **(**The Schedule for Affective Disorders and Schizophrenia for School-Aged Children) and a semi-structured severity rating, CYBOCS (Children’s Yale-Brown Obsessive-Compulsive Scale), and were in current psychiatric OCD treatment. They all had close contact to their psychologist and/or psychiatrist. This group will be referred to as the clinical group (CG). During April–May 2020, a questionnaire was sent to a total of 101 children and adolescents in CG. A total of 65 (65%) children and adolescents responded to the questionnaire.

The second sample of children/adolescents (7–21 years) were identified through the Danish OCD Association. Members of the Association were derived from all over Denmark. They were diagnosed years ago and their primary treatment was completed. They did not necessarily receive current psychiatric treatment. This group will be referred to as the survey group (SG). During April–May 2020, a questionnaire was sent to approximately 600 members of the association, mostly adults. A total of 37 children and adolescents responded to the questionnaire.

All data was stored in REDCap (Research Electronic Data Capture) (https://projectredcap.org/resources/citations/).

### Questionnaire

For both the CG and the SG, a self-report questionnaire was developed for use in the present study. The questionnaire was designed as to measure clinical variables related to OCD. The questionnaires were comparable. However, in the questionnaire for SG, questions concerning demographic variables as well as questions concerning other aspects of well-being were included. This included perceived changes in quality of life that was assessed using a visual scale ranging from 0 (life quality is highly improved) to 100 (quality of life is greatly reduced). In both questionnaires, five questions were used to measure the change in OCD severity. These questions were based on the symptom severity questions of the Yale-Brown Obsessive-Compulsive Scale (Y-BOCS) that measure the time spent on obsessions and compulsions, the interference, distress, resistance, and experienced control [12, 13]. The OCD severity questions were rated on a 5-point Likert scale where − 2 indicated a large improvement, 0 indicated no change and 2 indicated a large increase in severity. The total score for the severity change ranged from − 10 to 10, with 0 indicating no overall change [11].. The children and adolescents in both groups were furthermore asked to report on worsening of anxiety and depressive symptoms and change in avoidance behavior.

Parents and participants older than 15 years from the CG gave a written informed consent that their answers could be used in research. Participants from the SG gave informed consent by answering the anonymous questionnaire. Parents of younger children were allowed to help them answer the questions.

### Information from files

Parents and participants older than 15 years from the CG also gave their written informed consent for the use of information from their patient file. Collected data included information concerning the OCD symptoms (phenotype, severity, treatment, treatment outcome, insight), co-occurring psychiatric disorders, psychiatric disorders in the family, and whether the child/adolescent always had had a tendency to worry and speculate.

### Statistical analyses

Associations between total OCD severity change scores and possible predictor variables were analysed in univariate analyses using linear regression models. Secondly, multivariate analyses were used to examine the association between the total OCD severity change scores and significant associations detected in the univariate analyses.

Statistical analyses were performed using STATA.

## Results

### Participants

A total of 65 (65%, mean age 14.9 (SD = 2.66) years, 24 (36.9%) males) and 37 (mean age 14.14 (SD = 2.79) years, 12 (33.3%) males) participants responded to the CG questionnaire and the SG questionnaire, respectively. Demographic data for the two groups are shown in Table 1. For the CG, average age of onset was 9.35 (SD = 3.16) years. OCD score at time of diagnosis was 27.69 (SD = 4.02). At baseline 32 (53%) reported a good to fair insight, 17 (28%) reported a moderate insight and 12 (20%) reported a poor insight. A total of 51 (83.6%) reported moderate to severe avoidance at baseline. A total of 41 received therapy, and 29 were treated with a serotonin reuptake inhibitor (SRI), ten with a neuroleptic and seven with ADHD medication. A total of 42 (64.6%) were diagnosed with at least one co-occurring psychiatric disorder and 24 (36.9%) had a 1st family members with one psychiatric disorder. For the SG, 19 (51.4%) reported to have OC symptoms less than 5 years, 20 (54.1%) described cleaning symptoms, and 18 (48.6%) described aggressive symptoms. A total of 25 (67.6%) were engaged in therapy, and 12 (32.4%) received medication.

**Table 1:** Demographic data for the clinical group (CG) and the survey group (SG)

	CG demographic data Baseline data *N* = 65	SG demographic data *N* = 37
Age (SD)	14.89 (2.66)	14.14 (2.79)
Gender (Male)	24 (36.9%)	12 (33.3%)
Age of onset (SD)	9.35 (3.16)	Less than 5 years *N* = 19 (51.4%) More than 5 years *N* = 18 (48.6%)
Baseline OCD severity score (SD)	27.69 (4.02)	
OCD cleaning	52 (80%)	20 (54.1%)
OCD Hoarding	29 (44.6%)	
OCD aggressive/sexual	57 (87.7%)	18 (48.6%)
OCD symmetry	57 (87.7%)	
OCD checking	43 (66.2%)	
Insight		
Good-fair	32(52.5%)	
Moderate-poor	29 (47.5%)	
Just-right feeling		
Yes	42 (64.6%)	
No	23 (35.4%)	
Doubt		
0–1	22 (26.4%)	
2–3	31 (58.5)	
Responsibility		
0–1	34(59.7%)	
2–3	23 (40.3%)	
Avoidance		
0–1	10 (16.4%)	
2–3	51 (83.6%)	
Therapy at the time of questionnaire	41 (63.08)	25 (67.6%)
SRI medication at the time of questionnaire	29 (44.6%)	12 (32.4%) (medication)
Neuroleptic medication at the time of questionnaire	10 (15.6%)	
ADHD medication at the time of questionnaire	7 (10.9%)	
Diagnosis of Anxiety	18 (27.7%)	2 (5.4%)
Diagnosis of Depression	1 (1.5%)	1 (2.7%)
Diagnosis of Psychosis	3 (4.6%)	0 (0.0%)
Diagnosis of ADHD	14 (21.5%)	5 (13.5%)
Diagnosis of Tic disorder	11(16.9%)	1 (2.7%)
Diagnosis of Planning difficulties	9 (13.9%)	0 (0.0%)
Diagnosis of Anorexia	2 (3.1%)	2 (5.4%)
Diagnosis of developmental disorder	7 (10.8%)	4 (10.8%)
Diagnosis of Autism	7 (10.8%)	4 (10.8%)
Family history of psychiatric disorder that have influenced the child	12(18.5%)	
Family problems that have influenced the child	16 (24.6%)	
1stdegree family anxiety	17 (26.2%)	
1st degree family depression	13 (20%)	
1st degree family psychosis	1 (1.5%)	
1st degree family ADHD	8 (12.3%)	
1st degree family tic disorder	4 (6.2%)	
1st degree family autism	4 (6.2%)	
1st degree family dyslexia	9 (13.9%)	
1st degree family anorexia	2 (3.1%)	
1st degree family one psychiatric disorder	24 (36.9%)	
1st degree family two psychiatric disorders	9 (13.9%)	

### Results from the COVID-19 questionnaires

Data from both groups are shown in Table 2 and Table 3. In the CG, *N* = 29 (44.6%) reported a worsening of their symptoms with a mean worsening of 3,21 (SD = 1.78). A total of 21 (32.3%) reported a worsening of anxiety, and 22 (33.8%) of depressive symptoms. Of these, 11 children and adolescents reported a worsening of both. A total of 12 (18.5%) reported increased avoidance behavior. In the SG, 27 (73%) reported a worsening with a mean worsening of 4.19 (SD = 2.62). *N* = 20 (54.1%) reported a worsening of anxiety and *N* = 16 (43.2%) reported a worsening of depressive symptoms.

**Table 2:** Symptom intensity, anxiety, depressive symptoms, and avoidance behavior related to COVID-19

COVID-19 questionnaire	CG data *N* = 65 (%), mean (SD)	SG data *N* = 37 (%), mean (SD)
Symptom intensity		
Increased severity	*N* = 29 (44.6%), 3.21 (1.78)	*N* = 27 (73%), 4.19 (2.62)
Unchanged severity	*N* = 24 (36.9%)	*N* = 3 (8.1%)
Reduced severity	*N* = 12 (18.5%) -3.42 (2.94)	*N* = 7 (18.9%), −3.71 (3.04)
Anxiety increased	*N* = 21 (32.3%)	*N* = 20 (54.1%)
Depressive symptoms increased	*N* = 22 (33.8%)	*N* = 16 (43.2%)
Avoidance behavior increased	*N* = 12 (18.5%)	
New OCD symptoms	*N* = 10 (15.4%)	
COVID-19 part of OCD	*N* = 10 (15.4%)	13 (35.1%)

**Table 3:** Association between the worsening of anxiety, depressive symptoms, avoidance behavior and COVID-19 related OCD symptoms, and symptom severity during COVID-19 crisis

	CG data Mean (95% CI)	*p*	SG data Mean (95% CI)	*p*
Univariate Regression Analyses				
Anxiety	No *N* = 44: −0.20 (−0.98–0.58)	*p* = 0.0001*	No *N* = 17: 0.53 (−1.68–2.73)	*p* = 0.010*
	Yes *N =* 21: 2.90 (1.63–4.18)		Yes *N =* 20: 3.90 [(.38–5.42)	
Depressive symptoms	No *N* = 43: 0 (− 0.90–0.90)	*p* = 0.0021*	No *N =* 21: 1.14 (0.33–2.61)	*p* = 0.037*
	Yes *N =* 22: 2.36 (1.21–3.52)		Yes *N =* 16: 3.94 (1.48–6.39)	
Avoidance Behavior	No *N* = 53: 0.42 (−0.44–1.27)	*p* = 0.03*	NA	
	Yes *N =* 12: 2.5 (1.27–3.73)			
COVID 19 symptoms	No *N* = 55: 0.22 (−0.52–0.96)	*p* = 0.0001*	NA	
	Yes *N =* 10: 4 (2.24–5.75)			
Multivariate Regression Analyses				
Anxiety	2.46 (0.96–3.95)	*p* = 0.002*	3.57 (3.27–3.87)	*p* < 0.001*
Depressive symptoms	1.61 (0.21–3.01)	*p* = 0.025*	3.41 (3.11–3.70)	*p* = 0.862
Avoidance behavior	0.73 (−1.02–2.49)	*p* = 0.41	NA	

*p < 0.05 (significant association)

In univariate analyses, in both groups there was a significant positive correlation between aggravation of anxiety and depressive symptoms and the experienced worsening of OCD (Table 3). *N* = 10 (15.4%) in the newly diagnosed CG experienced new OC symptoms describing that thoughts about COVID-19 became an integral part of their OCD. This was also positively correlated to aggravation of OCD. In a multivariate analysis, anxiety and depressive symptoms continued showing a significant positive correlation to worsening of OCD symptoms (Table 3).

### Combination of data from the CG questionnaire and from patient files

In a univariate analysis of the CG data, there was a significant positive correlation between total OCD severity scores on the COVID-19 questionnaire and the occurrence of OCD aggressive and sexual symptoms (2.62, 95% CI [0.43–4.82], *p* = 0.02). Family history of psychosis resulted in an inverse relation. The number of patients with a family history of psychosis was rather small. There was a trend to a positive correlation to the occurrence of OCD symmetry symptoms, and reduced insight at baseline, whereas there was an inverse correlation when the child attended therapy. There was no association to OCD contamination/cleaning symptoms (Table 4). In the multivariate analysis, the positive correlation to OCD aggressive and sexual symptoms and to reduced insight at baseline remained significant or close to significant (Table 4.) In the SG, a comparable positive correlation between total OCD severity scores and the occurrence of aggressive symptoms was shown (3.10, 95% CI [0.56–5.65], *p* = 0.018), but no other predictors tested were found significant in this group (Table 4).

**Table 4:** Predictor analysis. The relation between baseline characteristics and symptom severity during COVID.19 crisis

	CG, Univariate analysis coef, 95%CI, p	SG, Univariate analysis coef, 95%CI, p
Age	−0.09 (− 0.37–0.19,) *p* = 0.53	0.37 (− 0.13–0.88), *p* = 0.138
Gender		
Male	0.74 (− 0.81–2.29), *p* = 0.34	0.04 (− 2.93–3.01), *p* = 0.977
Female		
Age of onset	0.05 (− 0.19–0.30), *p* = 0.68	Less than 5 years *N =* 19
		More than 5 years *N =* 18
		0.51 (− 2.25–3.26), *p =* 0.71
Therapy		
Yes	−1.24 (− 2.77–0.29), *p* = 0.11	1.58 (−4.48–1.32), *p* = 0.28
No		
OCD severity at time of diagnosis	− 0.11 (− 0.30–0.08), *p* = 0.23	
OCD contamination/cleaning		
Yes *N* = 52	− 0.35 (− 2.22–1.53), *p* = 0.71	0.32 (− 2.44–3.09), *p* = 0.81
No *N* = 13		
OCD hoarding		
Yes *N =* 29	−0.82 (− 2.32–0-68), *p =* 0.28	
No *N* = 36		
OCD aggressive/sexual		
Yes: 57	2.62 (0.43–4.82), *p* = 0.02*	3.10 (0.56–5.65), *p* = 0.018*
No: 8		
OCD checking		
Yes 43	0.38 (−1.20–1.97), *p* = 0.63	
No 22		
OCDsymmetry		
Yes 57	1.63 (−0.63–3.88), *p* = 0.15	
No 8		
Just-right feeling		
Yes 42	0.03 (−1.55–1.60), *p =* 0.97	
No 23		
Insight baseline		
0–1: 32 (Good-fair)	1.21 (−0.31–2.73), *p* = 0.12	
2–4: 29 (Moderate-poor)		
Doubt baseline		
0–1 22	0.69 (−0.92–2.30), *p* = 0.39	
2–4 31		
Responsibility baseline		
0–1: 34	0.38 (−1.27–2.03), *p* = 0.65	
2–3: 23		
Avoidance baseline		
0–1 10	−0.84 (−2.92–1.25), *p* = 0.43	
2–3 51		
SRI	0.11 (−1.40–1.62), *p* = 0.88	
Psychiatric comorbidity any	1.24 (−0.30–2.78), *p =* 0.11	−0.51 (−3.29–2.27), *p =* 0.71
Diagnosis of Anxiety	0.28 (−1.40–1.96), *p* = 0.74	
Diagnosis of Depression	1.22 (−4.89–7.32), *p* = 0.69	
Diagnosis of Psychosis	0.91 (−2.67–4.49), *p* = 0.61	
Diagnosis of ADHD	−0.56 (−2.39–1.26), *p* = 0.54	
Diagnosis of Tic disorder	−0.31 (−2.31–1.70), *p* = 0.76	
Diagnosis of Planning difficulties	1.01 (−1.16–3.17), *p* = 0.36	
Diagnosis of Anorexia	1.75 (−2.58–6.09), *p* = 0.42	
Diagnosis of developmental disorder	1.50 (−0.89–3.90) *p* = 0.21	
Diagnosis of Autism	0.22 (−2.20–2.65) *p* = 0.85	
Familiy histoy of illnesses that affect the child	−0.16 (−2.10–1.78) *p =* 0,88	
1st degree family of anxiety	−0.13 (−1.84–1.58), *p =* 0.88	
1st degree family of psychosis	−5.89 (−11.82–0.04) *p* = 0.05*	
1st degree family of depression	−0.42 (−2.30–1.45) *p =* 0.65	
1st degree family of autism	1.01 (−2.11–4.13) *p* = 0.52	
1st degree family of tic disorder	−0.32 (−3.45–2.81) *p* = 0.84	
1st degree family of dyslexia	−0.28 (−2.46–1.89) *p* = 0.80	
1st degree family of ADHD	−0.06 (−2.35–2.23) *p* = 0.96	
1st degree family one psychiatric disorder	0.58 (−0.97–2.13), *p* = 0.46	
1st degree family two psychiatric disorder	−0.28 (−2.46–1.89), *p =* 0–80	
Tendency - worry	0.39 (−1.15–1.93) *p* = 0.62	
	**Multivariate analysis coef, 95%CI, p**	
Therapy OCDaggressive sexuel	−1.04 (−2.55–0.47), *p* = 0.17 2.86 (0.50–5.22)*p =* 0.018*	
OCD symmetry	0.29 (−2.19–2.77) *p =* 0.81	
Insight baseline	1.40 (−0.08–2.88) *p* = 0.06	
Any psychiatric comorbidity	0.83 (−0.70–2.36), *p =* 0.28	

*p < 0.05 (significant association)

The participants were grouped depending on the experienced change in OCD symptom severity (Table 5). In the CG group, those that experienced a worsening, showed a significant inverse relation between OCD severity change and age, age of onset, and a trend towards an inverse correlation to a family history of tic disorder (Table 5). There was a positive correlation to a family history of ADHD and a trend towards a positive relation to OCD aggressive and sexual symptoms, insight at baseline, a diagnosis of anxiety and a family history of anxiety. (*p* < 0.20). Using a multivariate model analysing the variables identified in the univariate analyses, the relation to age (coef − 0.29, *p* = 0.007), age of onset (− 0.22, *p* = 0.009), family history of tic disorder (− 3.20, *p* = 0.0001) and ADHD (coef 2.45, *p* = 0.005) remained significant. Thus, in the clinical group of children and adolescents (CG), who experienced a worsening of OCD symptoms during COVID-19, the worsening was most pronounced in the younger participants, in those with earlier age of onset, and with a predisposition to ADHD. On the contrary, participants with a predisposition to tic disorder in the family showed a lesser degree of deterioration. However, when only those that experienced worsening were examined in the SG, no significant predictors were found (Table 6).

**Table 5:** Predictor analysis in the CG group, who experienced a worsening of their symptoms. The relation between baseline characteristics and symptom severity during COVID-19 crisis

CG subgroup experiencing a worsening of symptoms during COVID-19	*N* = 29 Coef, p Univariate analysis	*N* = 29 Coef, p Multivariate analysis
Age	−0.43, *p* = 0.001*	− 0.29 *p =* 0.007*
Gender	−0.51, *p* = 0.49	
Age of onset	−0.29, *p =* 0.01*	−0.22, *p =* 0.009*
Therapy	0.4, *p =* 0.56	
Baseline severity	−0.03, *p* = 0.72	
OCDcontamination/cleaning	0.008 *p* = 0.99	
OCDhoarding	−0.48, *p =* 0.49	
OCDaggressive/sexual	1.83 *p* = 0.16	
OCDchecking	0.78 p = 0.28	
OCDsymmetry	0.60 *p* = 0.59	
Insight baseline	1.02 *p* = 0.14	
Diagnosis of Anxiety	1.05 *p =* 0.18	
1st degree family anxiety	1.21 *p =* 0.14	
1st degree family depression	−1.14 *p =* 0.12	
1st degree family tic disorder	−2.37 *p* = 0.068	−3.20 *p =* 0.001*
1st degree family adhd	3.54 *p* = 0.004*	2.45, *p =* 0.005*

*p < 0.05 (significant association)

**Table 6:** Predictor analysis in the SG group, who experienced a worsening of their symptoms. The relation between baseline characteristics and symptom severity during COVID-19 crisis

SG subgroup experiencing a worsening of symptoms during COVID-19	*N* = 27 Coef, p Univariate analysis
Age	−0.336, *p* = 0.09
Gender	0.222, *p* = 0.84
Therapy, current	−0.889, *p* = 0.42
OCD contamination/cleaning	−0.117, *p* = 0.91
OCD aggressive symptoms	1.233, *p* = 0.24
Comorbidity, any	−1.544, *p* = 0.13
OCD duration	1.617, *p* = 0.11

*p < 0.05 (significant association)

## Discussion

The present study examined the impact of COVID-19 crisis on a clinical population of children and adolescents newly diagnosed with and in current treatment for OCD, and on a population of children and adolescents identified through the Danish OCD Association, who had been diagnosed years ago and where the primary treatment had been completed. The two groups were comparable in their mean age and gender distribution. The clinical group was almost all newly referred to the specialized clinic, with only a few showing a more chronic course. 63% were in active psychiatric therapy and almost 45% received SRI treatment. In the survey group, 67.6% participated in some form of therapy and 32% described the use of medication.

In both groups several of the participants reported a worsening of their OCD symptoms, anxiety and depressive symptoms as well as the extent of avoidance behavior. The most severe worsening of all parameters was seen in the survey group. In both groups the change of total OCD severity score correlated significantly with the worsening of anxiety and depression and the extent of avoidance. Thus, compared to the clinical group, the survey group seems to be more vulnerable to a crisis like COVID-19. The certainty of having a direct connection to a specialized hospital clinic may have a protective effect. Thus, in the clinical group both the patients and their parents knew that they very easily could be in touch with their therapist and/or a children and adolescent psychiatrist. The finding of positive correlations between an experienced aggravation of anxiety/depressive symptoms and avoidance, and the experienced worsening of OCD are in line with a recent study conducted by Secer et al. [9]. The study showed a direct predictive effect of COVID-19 fear on OCD symptoms in youths and an indirect effect mediated through emotional reactivity, which in turn predicted avoidance and depression-anxiety. Furthermore, both avoidance and depression-anxiety had a significant predictive effect on OCD [9].

In both groups, the occurrence of baseline aggressive/sexual OC thoughts and rituals increased the risk of experiencing a worsening of OCD symptoms during COVID-19 as measured by the time spent on obsessions and compulsions, the interference, distress, resistance, and experienced control. We were unable to show an association to baseline OCD contamination/cleaning symptoms. One might hypothesise that the reduced contact to the surrounding world, and the fact that everyone around them thoroughly were washing hands and were using hand sanitiser might have served as a sort of inflicted avoidance to the posed danger of the surroundings. Thus, at the time of the questionnaire, the children may have felt protected against the immediate treat. On the contrary, under the circumstances where the media made daily reports on the number of severely ill or the number of persons, who died from COVID-19 infection (even younger persons), these youngsters may be more affected by aggressive thoughts or worries that their parents or their grandparents could become ill or even die. Children would be vulnerable of losing their primary caretakers. As such they may have felt constantly reminded on the potential life-threatening infection making the risk of losing the primay-care takers or grandparents seem high.

In a recent study, Tanir et al. [11] examined symptom severity and presentation before and during COVID-19 pandemic. Both the study of Tanir et al. and our study showed an aggravation of symptom severity. Apart from the overall severity, the focus of the studies differed, however. By comparing the symptom presentation before and during the pandemic, Tanir et al. showed an increased number of children and adolescents with cleaning thoughts and contamination rituals during the COVID-19 pandemic. They did not, however, relate the change in symptom presentation to symptom aggravation measured by the CYBOCS. In the present study, we examined symptom aggravation related to baseline symptom presentation. Baseline symptom presentation was chosen since symptom dimensions have been suggested remarkably stable over time at least in an adult sample [14]. As such, symptom dimensions have been suggested to be useful in studies of predictors of treatment response [14].

Insight at baseline showed a protective factor against OCD symptom worsening. Poor insight is defined as beliefs that are estimated to be probably true (DSM-V). In anxiety studies, the term “intolerance of uncertainty” has been viewed as a cognitive vulnerability implicated in the maintenance of anxiety symptoms such as worry and avoidance [15]. A definition was proposed as “an individual’s dispositional incapacity to endure the aversive response triggered by the perceived absence of salient, key, or sufficient information, and sustained by the associated perception of uncertainty” [15]. Furthermore, in a study of adults completing a self-report measure during the H1N1 pandemic (2009), it was shown that intolerance of uncertainty predicted a lower level of coping strategies and greater anxiety. The pandemic was perceived as more threatening [16]. Poor insight may imply a degree of uncertainty of a potential threat. It might be hypothesised that the children and adolescents characterised by a poor baseline insight might have an increased vulnerability to a potential treat and thereby be more prone to react with more worry and fear. This hypothesis is strengthened by the finding of an inverse relation between insight and OCD severity and impairment in a pediatric OCD sample [17]. Furthermore, our group has shown that poor insight is positively associated with comorbid conditions and negatively associated with gaining remission [18]. Selles et al. [19] has recently shown that even though insight did not predict a reduced treatment response, response rates still depended on the degree of post-treatment insight and avoidance behavior [19]. Poor insight may thus define a more vulnerable group of children and adolescents that may continue being at risk for developing renewed OCD symptoms in situations where they are experiencing a trauma or psychological pressure. This would be in line with the previous suggestions that poor insight might influence the ability of the patient to modify irrational beliefs [20].

Altogether, the present study examined how children and adolescents with OCD react towards COVID-19 pandemic. Their reactions seemed to be more intense if they did not have an immediate contact to a psychiatric system. In both groups, aggressive and sexual OCD symptoms, and poor insight at baseline were predictors of a poorer outcome both on OCD severity, and on the worsening of anxiety and depressive symptoms. Furthermore, examining the clinical group of children and adolescents who experienced a worsening of their OCD during the crisis, the worst change of total OCD severity was associated with early age of onset and a family history of ADHD, whereas children with a family history of tics experienced a smaller change. Early-onset OCD has been suggested to be a distinct subtype with a more frequent occurrence in males, being associated with a greater global severity of OCD, a more frequent occurrence of comorbid tic disorder and associated with a greater prevalence of OCD in first-degree relatives [21]. The present study supports the idea of a separate subunit, also supporting that this earlier onset group may be particularly exposed to a severe worsening of OCD. Based on the present study, it is not possible to establish a direct link between the COVID-19 and the aggravation of OCD in children and adolescents. However, in line with Secer et al. [9], the severity correlated with the increased experience of anxiety/depressive symptoms and avoidance behavior suggesting that also in the present study a relationship between the fear of COVID-19 and the experienced severity of the OCD symptoms may be important. Thus, both the direct threat of the infection, and the consequences of social distancing, social isolation and the constant focus on hygiene may affect children and adolescents in general, and maybe more if they have a psychiatric vulnerability [22].

## Conclusions

The study points towards an influence of the OCD phenotype, baseline insight suggesting a continued vulnerability, and a family history of psychiatric disorders.

### Strengths and imitations

To our knowledge, this is one of the first studies to examine the effect of COVID-19 in samples of children and adolescents with OCD. The study examines the effect in two different samples strengthening the conclusions. The response rate was acceptable, and the answers were mostly complete. A limitation is that the study for both groups included a self-rated non-validated questionnaire. Furthermore, the number of participants in both the clinical and the survey group was limited. Finally, the number of predictors in the SG was limited since we did not have access to patient files.

Based on the study, a direct link cannot be established.

## Supplementary information

**Additional file 1.** Questionnaire on COVID-19.

## Data Availability

The datasets generated and/or analysed during the current study are not publicly available due to Danish law. JBN and DH confirm to have full access to all the data in the study, and to take responsibility for the integrity of the data and the accuracy of the data analysis.

## Abbreviations

OCD: Obsessive compulsive disorder
ADHD: Attention deficit hyperactivity disorder
CG: Clinical group
SG: Survey group
REDCap: (Research Electronic Data Capture)
