# Supplementary material for: The immediate effect of COVID-19 pandemic on children and adolescents with obsessive compulsive disorder
Source: BMC Psychiatry. 2020 Oct 20;20:511. doi: 10.1186/s12888-020-02905-5 (PMC7573524; doi:10.1186/s12888-020-02905-5)
Supplement: Supplementary file 1 — Additional file 1. Questionnaire on COVID-19. [file 12888_2020_2905_MOESM1_ESM.pdf]

Questionnaire on COVID-19

Thank you very much for taking the time to complete this questionnaire.
Your input is greatly appreciated.

COVID-19 has changed daily life for many people. We know that a great many people feel pressured by the situation and that many may have heightened concerns about the epidemic.

In this questionnaire, we would like to ask you how the COVID-19 epidemic has affected you or your child. In this way, we hope to gain a better understanding of how an epidemic of this magnitude may affect people. We also hope to be able to improve support and treatment planning.

The answers will remain anonymized and will be used solely for research purposes.

The questionnaire includes questions about the OCD and questions about your well-being in general
To ensure that your participation is voluntary, we include a consent form for you to sign. You can withdraw your consent at any time.

Questions about obsessions and compulsions

Did your obsessions and compulsions change after the COVID-19 outbreak occurred in Denmark?	yes no

If yes, please describe how it was changed

Do you experience a change in how much time you spend on obsessions and compulsions after the COVID-19 outbreak in Denmark?	I spend much less time<br>I spend less time<br>Unchange<br>I spend more time<br>I spend much more time

Which obsessions/compulsions do you spend more time on now?
Describe:

Which obsessions/compulsions do you spend less time on now?
Describe:

Do your obsessions and compulsions disturb your daily activities more after the outbreak of COVID-19?	They disturb me much less<br>They disturb me less<br>Unchange<br>They disturb me more<br>They disturb me much more

Do you experience more discomfort because of your obsessions and compulsions now after the outbreak of COVID-19?	Much less discomfort<br>Less discomfort<br>Unchanged<br>More discomfort<br>Much more discomfort
Are you trying to fight your obsessions and compulsions to a lesser degree after the outbreak of COVID-19?	No, fight much more<br>No, fight more now<br>Unchanged<br>Yes, fight less<br>Yes, fight much less
When your obsessions are active, has it become more difficult to withstand your rituals?	No, much easier<br>No, easier<br>Unchanged<br>Yes, more difficult<br>Yes, much more difficult
Do you avoid more situations now compared to the time without COVID-19? The question concerns situations or things apart from those recommended by the authorities	Yes No

If yes, please describe which situations or things

Have you experienced new OCD thoughts or rituals after the outbreak?	Yes No

If yes, please describe the new obsessions and compulsions

Have you experienced that COVID-19 has become part of your obsessions and compulsions?	Yes No

<u>Questions about other symptoms</u>

Do you experience more anxiety or has your anxiety changed after the outbreak of COVID-19?	Much les anxiety<br>Less anxiety<br>Unchanged<br>More anxiety<br>Much more anxiety
Have you experienced new anxiety symptoms after the outbreak?	Yes No

If yes, please describe the new symptoms

Do you experience increased sadness after the outbreak of COVID-19?	No, much less<br>No, less<br>Unchanged<br>Yes, more<br>Yes, much more
Which factors have affected you in relation to the COVID-19 outbreak in Denmark? Check the box, if any.	I could become ill<br>Others could become ill<br>I could infect other<br>How will it end<br>When will it normalise<br>Not going to school<br>Not being with friends<br>Not doing leisure activities<br>Being together in the family

Questions about COVID-19

How close have you been to the infection?	I have been ill with COVID19<br>I have been in quarantine<br>Family who were ill<br>Family who were in quarantine<br>Friends who were ill<br>Friends who were in quarantine<br>Heard about the infection
How often do you listen to news about COVID-19+	Less than once daily<br>Once daily<br>Several times daily
How often do you read about COVID-19?	Less than once daily<br>Once daily<br>Several times daily
How often do you talk about COVID-19 at home?	Rarely<br>Daily<br>Several times daily
How often do you talk about COVID-19 with friends?	Rarely<br>Daily<br>Several times daily

Do you think that your parents are worried?	No, not worried<br>Yes, a little worried<br>Yes, worried a lot
Do you think that your siblings are worried	No, not worried<br>Yes, a little worried<br>Yes, worried a lot

Thank you very much for your help
